# People’s Perceptions about the Importance of Forests on Borneo

**DOI:** 10.1371/journal.pone.0073008

**Published:** 2013-09-09

**Authors:** Erik Meijaard, Nicola K. Abram, Jessie A. Wells, Anne-Sophie Pellier, Marc Ancrenaz, David L. A. Gaveau, Rebecca K. Runting, Kerrie Mengersen

**Affiliations:** 1 Borneo Futures Project, People and Nature Consulting International, Jakarta, Indonesia; 2 Australian Research Council Centre of Excellence for Environmental Decisions, Centre for Biodiversity and Conservation Science, University of Queensland, Brisbane, Australia; 3 Center for International Forestry Research, Bogor, Indonesia; 4 Durrell Institute for Conservation and Ecology, School of Anthropology and Conservation, Marlowe Building, University of Kent, Canterbury, Kent, United Kingdom; 5 Hutan/Kinabatangan Orang-utan Conservation Programme, Sandakan, Sabah, Malaysia; 6 Research Fellow, North England Zoological Society, Chester, United Kingdom; 7 Sabah Wildlife Department. Kota Kinabalu, Sabah, Malaysia; 8 School of Mathematical Sciences, Queensland University of Technology, Brisbane, Australia; Zoological Society of London, United Kingdom

## Abstract

We ascertained villagers’ perceptions about the importance of forests for their livelihoods and health through 1,837 reliably answered interviews of mostly male respondents from 185 villages in Indonesian and Malaysian Borneo. Variation in these perceptions related to several environmental and social variables, as shown in classification and regression analyses. Overall patterns indicated that forest use and cultural values are highest among people on Borneo who live close to remaining forest, and especially among older Christian residents. Support for forest clearing depended strongly on the scale at which deforestation occurs. Deforestation for small-scale agriculture was generally considered to be positive because it directly benefits people’s welfare. Large-scale deforestation (e.g., for industrial oil palm or acacia plantations), on the other hand, appeared to be more context-dependent, with most respondents considering it to have overall negative impacts on them, but with people in some areas considering the benefits to outweigh the costs. The interviews indicated high awareness of negative environmental impacts of deforestation, with high levels of concern over higher temperatures, air pollution and loss of clean water sources. Our study is unique in its geographic and trans-national scale. Our findings enable the development of maps of forest use and perceptions that could inform land use planning at a range of scales. Incorporating perspectives such as these could significantly reduce conflict over forest resources and ultimately result in more equitable development processes.

## Introduction

Striking a balance between economic development and maintenance of biodiversity is increasingly challenging in the face of climate change, rapid human population growth and concomitant demand for natural resources. Understanding trade-offs and synergies between these objectives is particularly urgent in tropical forest areas which are home to more than 1.2 billion people [1], comprise some of the most species-rich habitats in the world [2], [3], and are experiencing high levels of forest loss [4]. Relationships between the use of forest resources and economic development are complex. Income from resource extraction or forest conversion can drive local economic development [5], and higher national income can further stimulate forest loss by raising demand for agricultural land [6]. At the same time, forests provide many ecosystem services that are not currently valued in economic terms, and their loss can have significant negative impacts on health and livelihoods, especially among the rural poor for whom forests are often important safety nets [6]. Indeed, 90% of the world’s 1.2 billion people living in extreme poverty depend to some extent on natural forest resources [7]. These counteracting forces make it difficult to generalize about the impacts of deforestation and forest degradation on people living in forest landscapes [8], [9].

Because of the complexity of the various interactions between different types of land and forest uses, it is also difficult to say generally whether or not rural people welcome the changes brought about by deforestation and forest exploitation. Land use and land cover changes depend on the interactions between economic drivers, policies and quality of governance [10], [11], but, at least in the more democratically governed tropical countries, the opinions of rural people could inform the development and implementation of policies. Knowing more about people’s uses and perceptions of forests could lead to better planning at landscape and regional scales.

Debates between rural and indigenous forest-based people and various levels of government have also been sparked by plans for development of a “Green Economy” in many tropical countries. Steps aimed at reducing carbon emissions through avoided deforestation, or increasing carbon sequestration through reforestation, have generated a lively debate between local people, who are directly affected by the concomitant changes in land use, and the governments determining land use change [12]. Still, it is often unclear whether the community voices in these debates represent broader community opinion, or the vested interests of particular people within communities or civil society organizations.

The present study focuses on the island of Borneo, an area of high forest diversity and rapid land cover change [13], [14]. As part of a broader project on optimization of land use and wildlife conservation – the Borneo Futures project – we investigate how people perceive and use forests, and which social and environmental factors influence these perceptions. Our motivations for focusing on these perceptions are twofold. Firstly, these forest perceptions are a valuable source of information on local perspectives, knowledge and beliefs, and how these vary spatially over the entire island. Focusing on perceptions and how this influences people’s decision-making provides an alternative to more traditional approaches of determining economic values of community forest use [15]. Better understanding of local people’s perceptions could help inform and shape political agendas with regard to land use, sustainability, and people’s rights, and result in more equitable land use decisions and other societal processes. Secondly, perceptions about forest values could be considered proxies for the relative importance of forest ecosystem services, such as provision of timber and non-timber forest products (e.g., food), disease control, flood regulation, provision of energy and clean water, temperature control, and carbon sequestration [16]. For many of these services, it is difficult to quantify rates of provision of services and how they relate to forest management [17], [18]. This makes the effective incorporation of complex or intangible services in decision-making highly challenging. Perception-based assessments complement more traditional and economic valuations of forest services [15], [19].

Whereas previous forest use and perception have mostly been analysed at the village (e.g. [20], [21]–[23]), watershed (e.g. [24], [25]), industrial concession [26], or district level [27], the present study encompasses an area two or three orders of magnitude larger than earlier known studies: about one third of the 743,330 km^2^ island of Borneo. The obvious trade-off in targeting such a vast area is that our study provides broad information about variation in forest perceptions at a very large landscape level, rather than deep insights into particular use and perception patterns at a single location or in much smaller areas. Our approach has the advantage of allowing us to translate perceptions about forests and their underlying socio-economic and environmental variables into information that informs political decision-making at a regional level. Understanding how these perceptions vary across large landscapes, for example, between their source, sink and use areas, and across different groups of human beneficiaries (*sensu* [28]), facilitates the prediction of deforestation impacts on forest service users, and might also help the prediction of future deforestation patterns.

We asked the following three questions to further our understanding of forest use and perceptions and their variation across Borneo: (1) What are villagers’ perceptions of (i) the value of forest uses (including direct use of forest products, and other economic or non-economic uses); (ii) the value of ecosystem services (cultural and spiritual importance, importance for health, direct health benefits and environmental health benefits); and (iii) advantages and disadvantages of forest clearing (for small-scale clearing and large scale clearing); (2) What socio-ecological and environmental factors are associated with these perceptions, at three different levels: (i) individual respondents; (ii) village demographics; and (iii) land use in landscapes surrounding the villages?; (3) Are there differences in perceptions across the study regions?

## Methods

### Ethics Statement

The interview surveys were conducted in 2009 when only The Nature Conservancy (TNC) was involved as implementing organization. Because TNC does not have a specific institutional review board or ethics committee, the interview survey approach was reviewed and approved by TNC’s social science specialist. We had written approval for our interview survey from the Indonesian Directorate General of Forest Protection and Nature Conservation and from the Sabah Wildlife Department to conduct the surveys in the Kalimantan provinces and in Sabah. Researchers of the University of Queensland (UQ) became involved from 2011 onward when we started to apply new statistical techniques to analyze the data. We did not obtain confirmation from UQ’s ethical review board, because by that time the interviews had already been conducted. Before beginning the survey, potential participants in the surveys were informed of the goal of the interviews through a statement read by the interviewer and assured that the data would be analysed anonymously (see Supplementary Information in reference 30 for details of this statement). Interviews were conducted following verbal consent of a potential respondent to participate.

### Primary Survey

**Figure 1:**
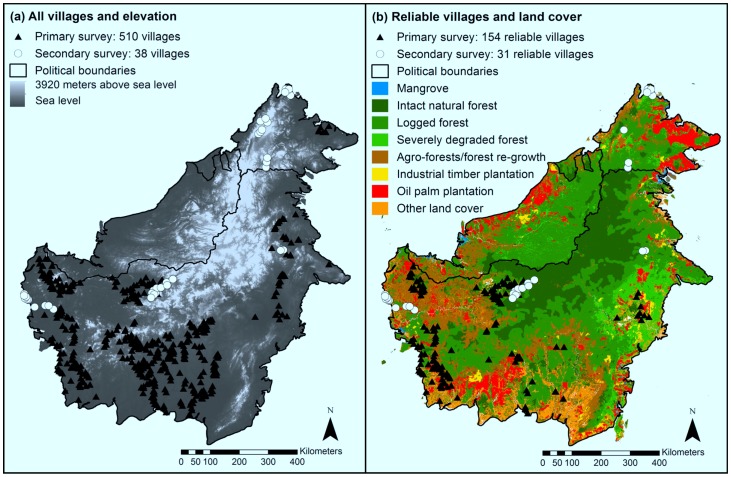
Location and geographic context of villages sampled in primary and secondary surveys in Indonesian and Malaysian Borneo. (a) All villages surveyed, overlaid with elevation information (DEM at 1 km^2^ resolution). (b) Villages with the most reliable information, overlaid with the 2010 land cover classes used in spatial analyses.

The primary dataset used for this study was extracted from a large interview survey conducted in Kalimantan, the Indonesian part of the island of Borneo, with a goal to better understand the social and environmental context of orangutan (*Pongo pygmaeus*) conservation [29], [30]. This initial survey was conducted by 19 local non-governmental organizations (NGOs) over a period of 15 months from April 2008 to September 2009, and involved interviews with 6,983 people in 687 villages within the general distribution range of orangutan in Kalimantan. All interviewers were selected based on experience with conducting surveys among local communities and fluency in the local languages, and were trained for this project. The original dataset of 6,983 interviews was reduced to 4,973 (see below under Survey data management), because we had doubts about the reliability of some of the responses [29]. A further 56 interviews were performed in six villages in the Malaysian State of Sabah (see Figure 1 for locations of primary survey villages). Village and respondent selection is described by Meijaard et al. [30]. The survey questionnaire comprised 32 questions and 34 optional sub-questions that were divided into a number of sections focusing on basic socio-demographic information, assessment of interviewee reliability, and questions on perceptions of forest values and wildlife.

The basic information at the respondent level included questions about the respondent’s age, sex, ethnic group, years of residence in the village and religion (Christian, Muslim, other). Respondents were also asked about the frequency with which they entered the forest (never; less than once a year; once or twice per year; once or twice per month; once or twice per week; two to four times per week; more than four times per week), and the reasons for entering the forest (logging; hunting; artisanal mining; collecting non-timber forest products other). Following Meijaard et al. [30], we used the number of forest trips over the past year (FT), and estimated the number of days each respondent spends in the forest each year. The scaled values were as follows: 4 trips/week: FT = 260; 2–4 trips/week: FT = 156; 1–2 trips/week: FT = 78; 1–2 trips/month: FT = 18; 1–2 trips/year: FT = 2; 1 trips/year: FT = 1; and 0 trips: FT = 0.

Direct economic uses of the forest were ascertained by directly asking the respondent, “what economic benefits do you obtain from the forest?” and asking directly whether or not these included each of timber, rattan, *gaharu* or aloes wood (*Aquilaria malaccensis*), honey, artisanal mining (mostly for gold, zircon, and gem stones), hunting, and traditional medicine. Other economic and non-economic forest uses were ascertained from two questions, about other economic benefits of the forest, and other reasons the respondent entered the forest. Cultural and spiritual importance was ascertained from the multiple-choice question, “does the forest play a significant cultural and spiritual role in your and your families lives”, with answers expressing the level of importance or “don’t know”. Importance for health was ascertained from a similar multiple choice question, “what is the importance of the forest for the health of you and your family” (“very important”, “quite important”, “not important”, and “don’t know”). Answers to an open-ended follow-up question, “what is the reason for the importance of the forest for health” were given as text and were divided into Direct health benefits and Environmental health benefits. The three measures of perceptions about forest clearing were obtained from a direct question, “does forest clearing provide benefits to you and your family” (“yes”; “no”; or “don’t know”) and a follow-up open-ended question about the reason for the interviewee’s opinion on forest alteration. Meijaard et al. provide a detailed overview of the questionnaire design (see supplementary information in [30]).

### Secondary Surveys

Sampling design of villages was originally oriented towards orangutan distribution areas within or near forest, hence resulting in a potentially biased sample with respect to the aims of the present study. To reduce this bias, we conducted post-stratified secondary surveys across the whole region of interest, based on geographic, social, ethnic and religious variations (see Figure 1a). These surveys included additional villages within previously surveyed regions in West Kalimantan and previously un-sampled areas within the West and East Kalimantan Provinces (236 respondents) and Sabah (145 respondents).

The secondary Kalimantan surveys employed the same questionnaire as the primary surveys but with a set of additional questions for more in-depth analysis. These surveys were conducted by one person (author ASP) with good Indonesian language skills in collaboration with local assistants. Interviewee selection and other methods equalled those of the primary surveys. The secondary Sabah survey employed a reduced set of questions focusing on forest use and perceptions only. These surveys were conducted by a team of nine Malaysian field research assistants from the local NGO HUTAN. In Sabah we specifically selected 15 villages that would encompass different ethnic group and religious identities as well as areas with differing histories of deforestation and dominant land-uses (Figure 1b).

### Survey Data Management

Text answers were recoded by the project lead (EM) who had not been directly involved in the interviews. Every text entry for forest goods and services was assigned a coded value, except in those cases where the types of goods/services were considered very similar, in which case they were merged into one category. Not all interview teams conducted the questionnaires with the same level of diligence and consistency. Consequently, we ascertained response reliability by measuring response patterns from each village and corresponding NGO based on text lengths, content and variation of ‘open’ question responses. In many cases, interview teams had not answered perception questions at all or had given the same answer for all respondents in a village, either indicating that the question was asked in a group rather than individual context, or that data were not appropriately recorded for each respondent. On the basis of these assessments, author EM assigned a reliability score to each village of: ‘1’ if no responses had been recorded, responses were of poor quality, or were apparently duplicated within one village; ‘2’ if good quality, i.e., answers had been genuinely reported but not much information was provided; and ‘3’ if excellent quality, i.e., detailed responses were reported for each individual respondent. We used data deemed as good and excellent in quality, reducing the dataset to 1,837 respondents from 185 villages (see Fig. 1 for the locations of these 185 villages). By choosing an evaluator independent of the primary analysts, we distanced the process of analyzing the data from the process of deciding on ‘reliable’ respondents.

### Village Level Variables

For each village included in the surveys we collected information on the history of the village (year of establishment), total population size, number of men, number of women, percentage of villagers who are Muslim, Christian or adhere to other religions, number of schools, presence of customary forest land, main sources of village income (oil palm, coconut, rice, rubber, cacao, pepper, vegetable, hunting, mining, fishing, and non-timber forest products), presence of industrial scale land users (timber, plantations, mining), and history of natural disasters (floods and landslides).

### Land use and Land Cover Variables

Land use and land cover (LULC) variables were developed to assess relationships between people’s use and perception of forests and the landscape contexts of their villages. Spatial data layers were developed for all of Borneo for eight LULC types: mangrove; intact natural forest; logged forest; severely degraded logged forest; agro-forests/forest re-growth; industrial timber plantations; oil palm plantations; and, other land cover types (see Table 1 for brief descriptions). The LULC data were derived from the integration of three principal datasets: (1) a SarVision PALSAR 2010 (50 m resolution) classification, where classes were used individually or aggregated together to form more generic classes (see Table S1 for details); (2) a road density index layer, used to distinguish intact, logged and degraded forests, was developed using digitized 1990–2000–2010 logging road network data (indicating mechanized logging) and transformed into a road density index (km/km^2^) for 1×1 km grid cell (search radius 5 km) using ArcGIS 10 (DG unpubl. data); and (3) digitised datasets of oil palm and industrial timber plantations, developed through onscreen digitising (using ArcGIS 10) of >150 Landsat images from 1990-, 2000-, and 2010-eras, downloaded from the Global Land Survey database (http://earthexplorer.usgs.gov/) (for details see [31], [32]).

**Table 1:** Outline of the eight land use/land cover classes used in the spatial analysis.

Land cover/Land use classes	Brief description
Mangrove (Mangrv)	Closed canopy Medium Forest with closed canopy of 10% to 30% occurring in tidal affected zones.
Intact natural forest (Intact)	Various types of old-growth natural forests that have never been logged by the timber industry.
Logged forest (Logged)	Various types of old-growth natural forests that have been logged by the timber industry using heavy machinery and networks of logging trails.
Severely degraded logged forest (Svlog)	Various types of old-growth forests that have become so severely degraded by logging and fire that they no longer resemble the spectral signatures of forests.
Agro-forests/forest re-growth (Agroreg)	Medium to tall agro-forests and forest re-growth including traditional rubber agro-forests, fruit gardens, and land under fallow, where forests are regenerating.
Industrial timber plantation (Indtim)	Planted or recently cleared industrial scale timber plantations, as of year 2010.
Oil palm plantations (Oilpalm)	Planted or recently cleared industrial scale oil palm plantations, as of year 2010.
Other land cover (Otherlc)	Includes various low-canopy shrubby vegetation types.

To calculate LULC variables for each village sampled, we initially mapped the localities within a Geographic Information System (ArcGIS 10). This was done by using the GPS (Global Positioning System) way-point taken at the centre of each of the sampled villages. We then used a buffer tool to calculate concentric circles (buffers) of 3, 5, 10 and 20 km radii from each village location point. The tree kilometre radius was used to account for immediate land cover type in villages which often stretch for several kilometres, while the 5 km radius was chosen as this would capture environmental variables very close to the village. The 10 and 20 km values were chosen because these gave a wider overview of adjacent land cover types surrounding these villages and hence potentially influencing people’s perceptions. These buffers were then overlaid with the eight LULC layers in vector format, and for each buffer class the eight variables were extracted. The areas of the land classes were measured to the nearest hectare for each buffer and the percentage cover of the class was then calculated. We incorporated the percentage values (in each radius) in the spatial analysis described below to help understand how people’s usage of forest products and the perceptions of forest benefits may differ with varying land use and land cover around their villages.

### Statistical Analysis

#### Characterisation of perceptions

To answer the first question posed in the Introduction, the following indices were constructed from questionnaire response items: Direct economic uses (7 items); Other forest uses (29 items); Cultural and spiritual importance (1 item); Importance for health (1 item); Direct health benefits (11 items, e.g., medicines, disease prevention); Environmental health benefits (14 items, e.g., flood prevention, clean air, source of water); Advantages of small-scale clearing (11 items); Advantages of large-scale clearing (10 items); and Disadvantages of large-scale clearing (22 items). Indices that comprised only one item (Cultural and spiritual importance, Importance for health) were coded as binary (1 = important; 0 = not important or don’t know). The Direct economic uses index was constructed as a weighted average of the seven specified items, scaled to lie between 0 and 1. We assigned different weightings (a multiplier of 1, 2, or 3) to different forest products based on our knowledge of relative economic importance of the seven different forest products in village economies and assessments of some 25 studies in the published literature (e.g., [33], [34], [35]): timber (3); rattan (2); *gaharu* (2); honey (1); artisanal mining (3); hunting (3); and traditional medicine (1). The relative value of these products varies significantly between villages, and without a formal analysis of what drives this variation we admit that our weighting is informed but somewhat subjective. The alternative approach of assigning equal weight to these products is similarly problematic and we believe our weightings add realism to the study. Each of the other indices was constructed as a scaled unweighted sum of responses to open-ended questions in the survey, where responses were coded as a set of binary items indicating whether the subject had or had not nominated that item in their open-ended response, and scaling was to the range 0–1. For example, the index of Direct health benefits had raw values ranging from 0 to 11, with the former indicating that the subject had nominated no direct benefits and the latter indicating that the subject had nominated all of the 11 benefits recorded in any of the interviews. This total was then scaled to a maximum of 1, to give an index in the range 0–1 for the analyses. The final index, Ecosystem Services, was constructed as the sum of the indices for Cultural and spiritual importance, Direct health benefits and Environmental health benefits, then scaled to lie between 0 and 1.

#### Socio-ecological factors associated with perceptions

In line with the second study question, three groups of explanatory variables were used in the statistical analyses: (i) individual level covariates; (ii) village level covariates; (iii) land cover covariates. The individual level covariates included gender, age, religion, ethnic group and time spent in the forest (FT). The village level covariates included population size (number of people in village), schools per individual, primary ethnic group, religion (% Christian, % Islam, % other). The land cover covariates included percentage of land cover in each of eight types (mangrove; intact natural forest; logged forest; severely degraded logged forest; agro-forests/forest re-growth; industrial timber plantation; oil palm plantations; and other land cover types) within a series of circular areas around the village (of radii 3, 5, 10, 20 km).

We examined relationships between the seven direct economic forest uses and the available socio-ecological variables using Classification and Regression Trees (CART) and Boosted Regression Tree (BRT) analyses. In a CART analysis, the response variable is described by a cascading series of binary splits of the explanatory variables; this is often represented as a tree-like structure with the final nodes representing homogeneous subsets of the responses. The selection of variables, the placement of the variables in the tree model, and the choice of location of the binary split are all data-dependent and determined by the model. A Boosted Regression Tree (BRT) analysis is a boosted form of CART, in which many shallow trees, based only on the primary splits, are formed on random subsets of the data and then combined. Whereas CART analyses provide explicitly interpretable models of interacting sets of predictor variables to predict a response, BRT and its analogues have been shown to provide improved predictive performance [36].

The BRT models were fitted using the function ‘gbm.step’ for generalized boosted regression models in the ‘dismo’ package [37] in the ‘R’ environment for statistical computing, version 2.15.0 [38]. The BRT models were fitted with the following specifications: a continuous response with a Laplace (absolute deviation) loss function, 5,000 trees with an interaction depth of 2 (including 2-way interactions), bagging fraction of 0.5 (i.e., 50% random samples used for fitting the trees), training fraction of 0.8 and five-fold cross-validation. The performance of the model was also assessed using five-fold cross-validation and the adequacy of the choice of the number of trees was confirmed. The CART models were fit using the ‘R’ package ‘rpart’ [39] and were based on 5-fold cross validation with strict cost-complexity measures (cp = 0.015, minsplit = 100, maxdepth = 5).

#### Spatial consistency of forest perceptions

The third study question was addressed by testing for equality of the index values across provinces and across survey datasets. These tests were conducted using analyses of variance for the continuous indices and logistic regression for the binary indices.

#### Sensitivity analyses

Sensitivity analyses were conducted to assess the robustness of the statistical inferences to various modelling choices. The sensitivity analysis comprised three main evaluations. (1) The choice of classification of the indices (as None, Low, Medium, High) was assessed. The indices were fitted in two alternative ways, first as a continuous variable, and then dichotomised as a binary variable indicating any positive response versus no positive response. (2) The choice of statistical model was assessed. Three alternative models, namely generalised linear regression (GLM) and generalised additive model (GAM) regression, were fitted. The GLM analysis was also extended to a mixed model, to account for respondents within villages; the GAM analysis further extended this to allow for nonlinear relationships between the explanatory variables and the response. All models were fitted using the three representations (continuous, categorical, binary) of the seven indices as responses and the three sets (representing individual, village and forest) of explanatory variables, as described above. (3) The impact of restricting the dataset to high quality responses was assessed by applying the analyses to the entire dataset of survey respondents.

## Results

### Respondent Demographics

Based on all valid survey responses, the respondent were 89% male and 11% female; 66% of Dayak origin (collective name for indigenous ethnic groups, mostly from the interior of Borneo), 17% primarily coastal origin (Malay, Banjar and Kutai people), 17% immigrants (Javanese, Balinese, Buginese and others), and <1% formerly nomadic people (Punan and Orang Ut); and 45% Muslim, 44% Christian, and 11% other religions (Buddhist, *Kaharingan*, Hindu). Of the 1,837 respondents included in the analyses of forest perceptions, the gender composition was 79.5% male and 20.5% female; the ethnic composition was 50% Dayak origin, 34% coastal and 15% immigrants; and the religious composition was 53% Christian, 45% Muslim, and 2% other religions. The gender bias was caused by the initial focus of the interview studies on orangutans, about which men were more knowledgeable, and therefore more often selected for interviews (see [30]).

### Characteristics of Forest Perceptions

#### Forest perception indices

**Figure 2:**
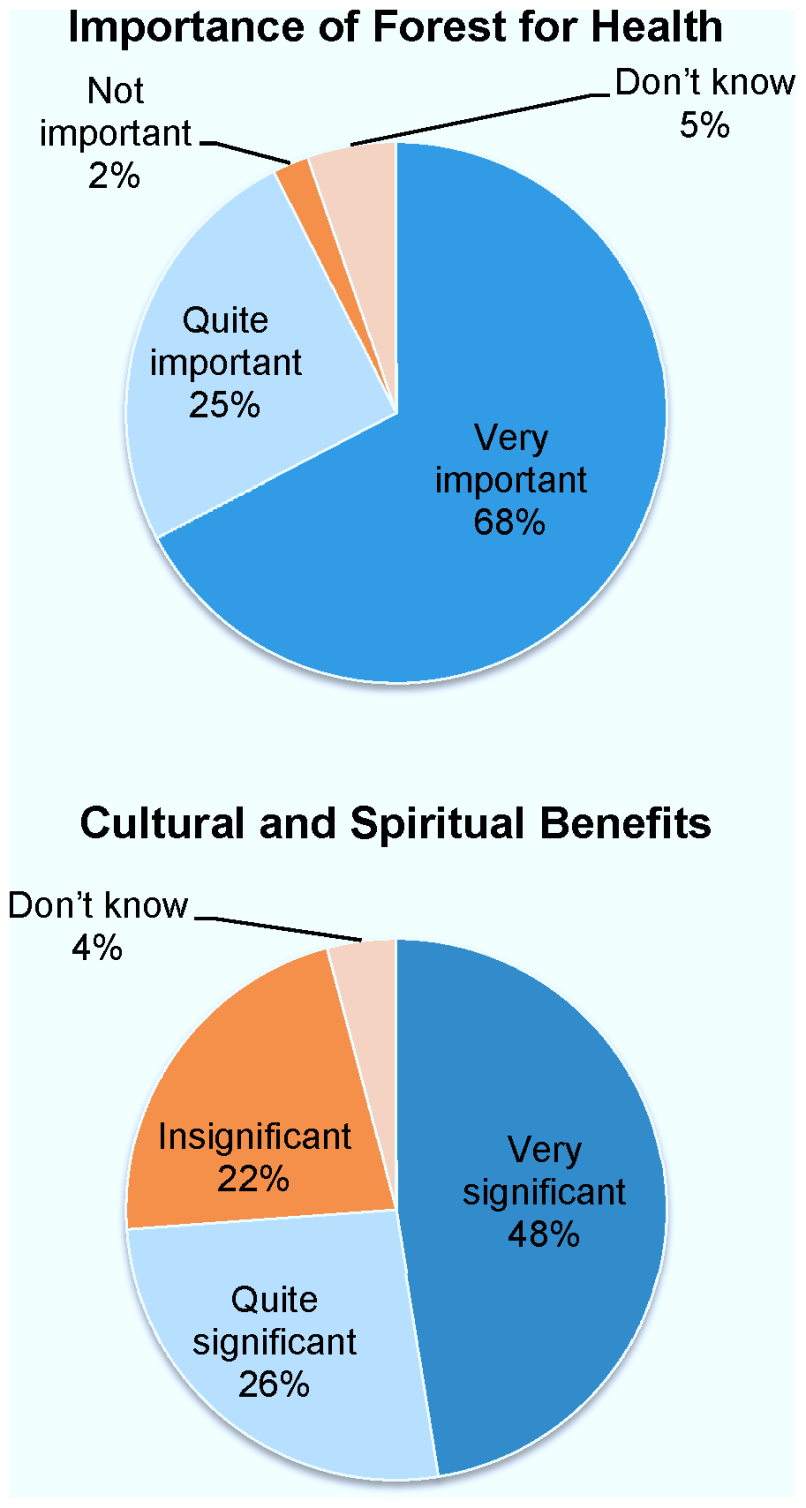
Perceptions on the Importance of Forest for Health and for Cultural and Spiritual Benefits expressed as percentages of the total of respondents’ answers. (a) Importance of Forest for Health. (b) Cultural and Spiritual Benefits. Analyses are based on the full data set.

Perceived values of forests were generally high, with especially the importance of forests for health (Figure 2a) and cultural and spiritual purposes (Figure 2b) emphasized by the respondents’ replies. Also, perceived environmental health benefits were high with 65% of all respondents volunteering one or more benefits. These patterns were reflected by the number of responses given for people’s perceptions about the advantages of small-scale deforestation, advantages of large-scale clearing, and disadvantages of large-scale clearing (respectively 990, 586, and 1,190 responses among reliable respondents, see Text S1, which summarizes the coded responses given by individual respondents). Statistical summaries of the ten indices are given in Table S2, with the items that contributed most strongly to the variation in respondent scores for each index shown in Table 2.

**Table 2:** Dominant items in the forest perception indices, based on Analysis of Variance (for further details see Text S1).

Index	Dominant factors
Direct economic uses	Timber, mining, hunting
Other forest uses	Fish, illipe nuts, forest gardens, rubber
Direct health benefits	General welfare, medicine
Environmental health benefits	Cool shade, source of water, clear air, flood prevention
Advantages of small-scale clearing	Needed for forest gardens, agricultural crops, rubber
Advantages of large-scale clearing	Good for work or business, a source of income
Disadvantages of clearing	Negative impacts on the community, income, environment, and forest products

Correlations between eight of the indices are small (Table 3), suggesting that the indices are measuring different aspects of perceptions on the importance of forest for livelihoods and health (note, the two indices for Cultural and spiritual importance and Importance for health, were excluded since they are binary). The largest correlation (r = −0.42) indicates that respondents who nominated a larger number of Environmental health benefits (e.g., cooling effect of forests, water retention, flood prevention) tended to nominate a smaller number of Direct health benefits (e.g., medicinal plants), suggesting that individuals tended to focus their answer either on direct or environmentally-mediated health benefits, not both. As we will highlight in the Discussion, perceptions of Environmental health benefits were particularly strong in areas where those benefits are dwindling (deforested areas), whereas perceptions of Direct health benefits were strongest in forested areas.

**Table 3:** Pearson correlations (*r*) between indices, with moderately large values in bold,: (1) Direct economic uses; (2) Other forest uses; (3) Environmental health benefits; (4) Direct health benefits; (5) Ecosystem services (sum of indices 3,4 and Cultural and spiritual importance); (6) Advantages of small-scale clearing; (7) Advantages of large-scale clearing; (8) Disadvantages of large-scale clearing.

	(2)	(3)	(4)	(5)	(6)	(7)	(8)
**(1)**	**0.24**	−0.06	0.19	**0.26**	0.08	0.05	0.10
**(2)**		0.004	0.07	0.11	0.006	0.12	0.14
**(3)**			**−0.42**	0.11	−0.12	0.06	0.11
**(4)**				0.20	0.06	−0.01	0.06
**(5)**					0.02	−0.03	0.05
**(6)**						−0.05	**−0.25**
**(7)**							−0.16

The next largest correlations indicate a positive associations between Direct economic uses and each of Other forest uses (*r = *0.24) and Ecosystem services (i.e., sum of both Environmental health and Direct health benefits, and, Cultural and spiritual importance) (r = 0.26), suggesting that all these indices were influenced by the same factor.

Finally, we point out the negative association between the scores for Advantages of small-scale clearing and Disadvantages of large-scale clearing (*r* = −0.25), which indicates that people in areas with significant small-scale agricultural activities (mostly shifting cultivation) generally had negative perceptions about the impacts of large-scale land clearing (mostly plantation development).

### Socio-ecological Factors Associated with Forest Perceptions

Different sets of variables were identified as most important for the different indices (Tables 4 and 5). Some indices were dominated by a single variable; for example, Direct economic uses was strongly explained by the religious composition of the village. Most indices were explained by a combination of variables. This diversity of description is consistent with the observation mentioned earlier that the indices appeared to be measuring different aspects of the respondents’ perspectives of the forest. Overall, religion, ethnicity, age of respondent, and village population size were consistently important, although other factors such land cover, the province in which the villages were located, and the number of years a respondent had lived in the village also played important roles (Tables 4 and 5).

**Table 4:** The ten most important explanatory variables for the Direct economic uses, Other forest uses, Cultural spiritual importance, Importance for health, and Environmental health benefits indices in the BRT analysis, showing the explanatory variables in order down each column with their relative importance.

Direct economic uses		Other forest uses		Cultural spiritual importance		Importance for health		Environmental health benefits	
Vill.Relig.	22.9	Population	15.4	Tribe	10.2	Age	8.7	Tribe	10.9
Pop’n	9.8	FT	12.5	Vill.Relig.	9.1	Res.Yr	8.1	Vill.Relig.	9.0
FT	6.2	Pop’n	10.0	Religion	8.8	Vill.Relig.	7.8	Rubber	7.8
Province	4.6	Res.Yr	7.2	Age	8.2	Intact5	7.3	Religion	7.8
Rice	3.4	Vill.Relig.	5.4	Rubber	8.0	Agroreg20	7.2	Age	7.1
Res.Yr	3.2	Fishing	5.0	Province	6.4	Mangrv20	7.2	Province	6.1
Age	3.1	Agroreg20	3.7	Pop’n	5.5	Indtim10	5.3	Pop’	4.9
Agroreg10	3.0	Logged20	3.7	Agroreg20	4.0	Oilpalm3	4.8	FT	3.9
Agroreg5	2.9	Province	3.5	FT	3.0	Agroreg10	4.1	Res.Yr	3.6
Hunting	2.9	Otherlc20	3.4	Mangrv20	2.9	Intact3	3.7	Agroreg20	3.5
*r = 0.82*		*r = 0.70*		*Acc. = 0.89*		*Acc. = 0.90*		*r = 0.48*	

Hunting, Rice, Rubber, Fishing refer to predominant activities of the village. Religion refers to individual respondent’s religion. Vill.Relig. refers to composition of religion in the village. Age = age of respondent. FT = time spent in the forest (see Methods). Res.Yr = number of years the respondent has lived in the village. Pop’n = number of individuals per village. Schools = number of schools per village.

* For abbreviations of land use classes and area radius, e.g. Agroreg5, see Table 1.

**Table 5:** The ten most important explanatory variables for the Direct health benefits, Ecosystem services, Advantages small scale clearing, Advantages large scale clearing, and Disadvantages large scale clearing indices in the BRT analysis, showing the explanatory variables in order down each column with their relative importance.

Direct health benefits		Ecosystem services		Advantages small scale clearing		Advantages large scale clearing		Disadvantages large scale clearing	
Population	11.6	Vill.Relig.	21.1	Vill. Relig.	10.1	Res.Yr	18.2	Res.Yr	11.3
Res.Yr	10.6	Rubber	5.9	Province	9.5	Religion	11.7	Logged20	11.3
Vill.Relig.	7.8	Mangrv20	4.8	Pop’n	7.7	Oilpalm3	11.2	Religion	8.4
Logged20	6.6	Province	4.8	Logged10	7.4	Agroreg3	7.5	Pop’n	6.9
Age	5.6	Indtim10	4.6	Res.Yr	6.5	Pop’n	7.2	Agroreg10	6.2
Religion	5.5	Religion	4.5	Age	4.5	Province	6.5	Vill.Relig.	5.0
Logged10	5.4	Pop’n	3.5	Agroreg5	4.0	Oilpalm	4.8	Age	4.9
Agroreg10	3.8	Intact10	3.5	Indtim20	3.4	FT	2.6	Province	4.8
Intact20	3.2	Res.Yr	3.4	Religion	3.2	Vill.Relig.	2.6	Indtim5	4.3
Otherlc10	3.0	Age	3.4	Oilpalm	2.8	Schools	2.6	Oilpalm	3.3
*r = 0.49*		*r = 0.72*		*r = 0.66*		*r = 0.60*		*r = 0.5.9*	

For explanation of variables see Table 4.

**Figure 3:**
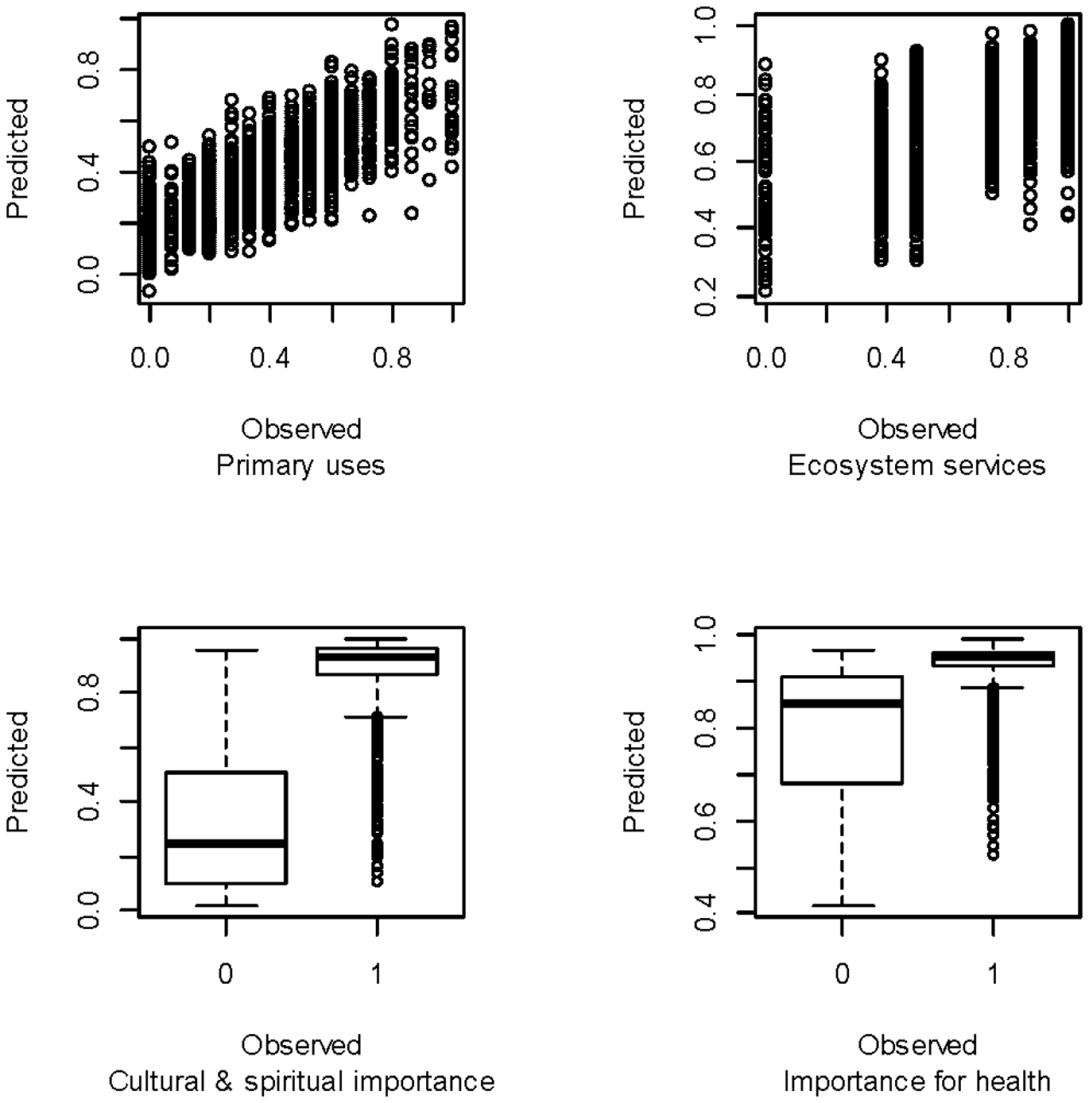
Observed versus predicted values for four indices, based on BRT analyses.

By comparing the observed and predicted index values using Pearson correlation coefficients for the continuous indices and overall % accurate classification (Acc.%) for the binary indices (Tables 4 and 5) we found that the model fits were generally good; the models explaining almost half of the total variation for some indices, and substantially more for the remainder (Figure 3).

The CART analyses confirmed the strongly interacting nature of the socio-ecological variables in predicting forest perceptions. In most cases, variables identified as most dominant in the BRT analyses were also important in the CART analyses; however, as anticipated, the two methods produced different combinations of important variables due to the near-equal importance of several of the variables, and the high frequency of interactions among variables (as indicated in Tables 4 and 5), that is, the effect of one variable depends on the levels of other variables. Moreover, the BRT analyses were based on an aggregation of many trees, whereas the CART analyses represented the results as a single tree. Note that both analyses naturally handle these interactions between variables.

**Figure 4:**
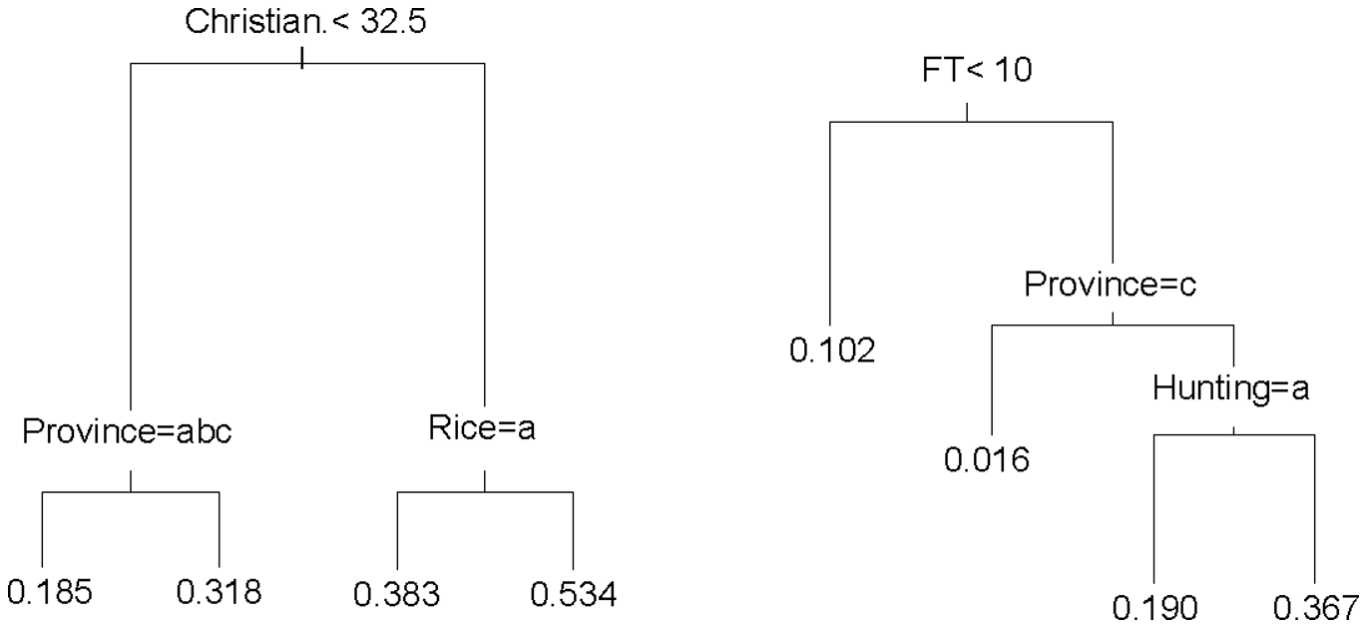
Regression trees for Direct economic uses and Other forest uses, based on CART analyses. (a) Direct economic uses. (b) Other forest uses. Each classification shows the value or threshold for taking the branch on the left side of the split below it. Values at the tips of the trees indicate the mean value of the index for that group of responses. The classifications are: Christian (% Christians in the village), Province (a = Central Kalimantan, b = East Kalimantan, c = Sabah, d = West Kalimantan), Rice and Hunting (a = no, b = yes as a predominant activity of the village), FT (index of time spent in forest); Population (population of the village).

Analysis of the regression tree for the Direct economic uses index (Figure 4) indicated that the dominant variable was religious composition of the village, followed by ‘Province’ and whether or not rice cultivation is a predominant activity of the village. The smallest average scores, indicating low levels of reported economic uses of the forest, were for respondents in villages with a smaller percentage of Christians in Central Kalimantan, East Kalimantan and Sabah. The largest average scores were obtained for those respondents in villages with a higher percentage of Christians and rice cultivation as a predominant activity of the village. For the Other forest uses index, the dominant variable was the amount of time that the respondent spent in the forest (FT), with further contributions from ‘Province’ and whether or not hunting was a predominant activity of the village (Figure 4).

**Figure 5:**
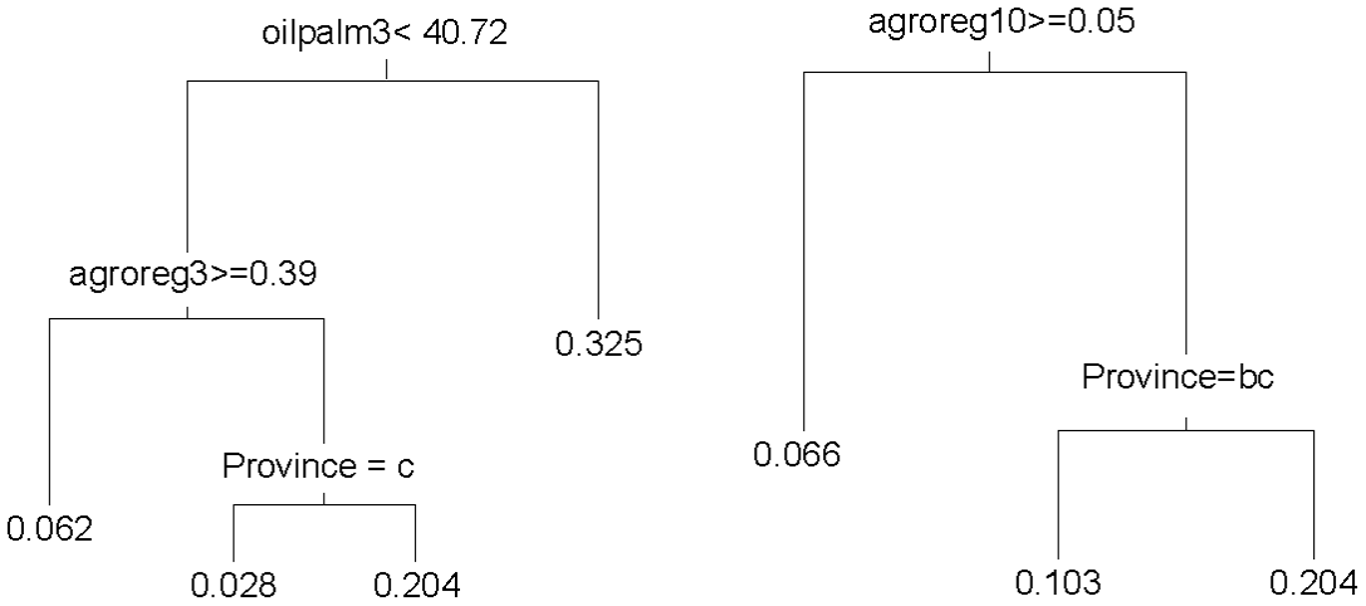
Regression tree for advantages and disadvantages of large-scale clearing, based on CART analyses. (a) Advantages of large-scale clearing. (b) Disadvantages of large-scale clearing. Classifications are explained in Figure 3 caption. Other classifications are oil palm3 (% area in a 3 km radius of the village that is oil palm), similarly for agroreg3 (agro-forests, forest regrowth, 3 km radius) and agroreg (10 km radius).

The CART analysis confirmed the difficulty of explaining variation in the Advantages of small-scale deforestation index (regression tree not shown). This index varied most strongly across provinces, with smallest scores for respondents in Central and East Kalimantan. The largest scores for Advantages of small-scale deforestation were obtained for respondents in Sabah and West Kalimantan, in villages with a large proportion (65%) of logged forest in a 10 km radius, and in villages with a larger number of families (>550). The model for the index regarding Advantages of large-scale deforestation comprised a strongly dominant variable (i.e., oil palm plantations at 3 km around the village), with other variables including ‘Province’ and ‘Agro-forests/forest re-growth’ (“agroreg”) at a 10 km radius determining secondary splits. Largest average scores were obtained for respondents in villages with substantial oil palm close to the village (Figure 5a). Opinions about Disadvantages of large-scale deforestation were most strongly influenced by education (represented by number of schools per individual), interacting with ‘Province’ and land cover (logged forest and Industrial timber plantation “Indtim”) at 20 km radius (Figure 5b).

### Consistency of Perceptions across Geographic Regions

**Figure 6:**
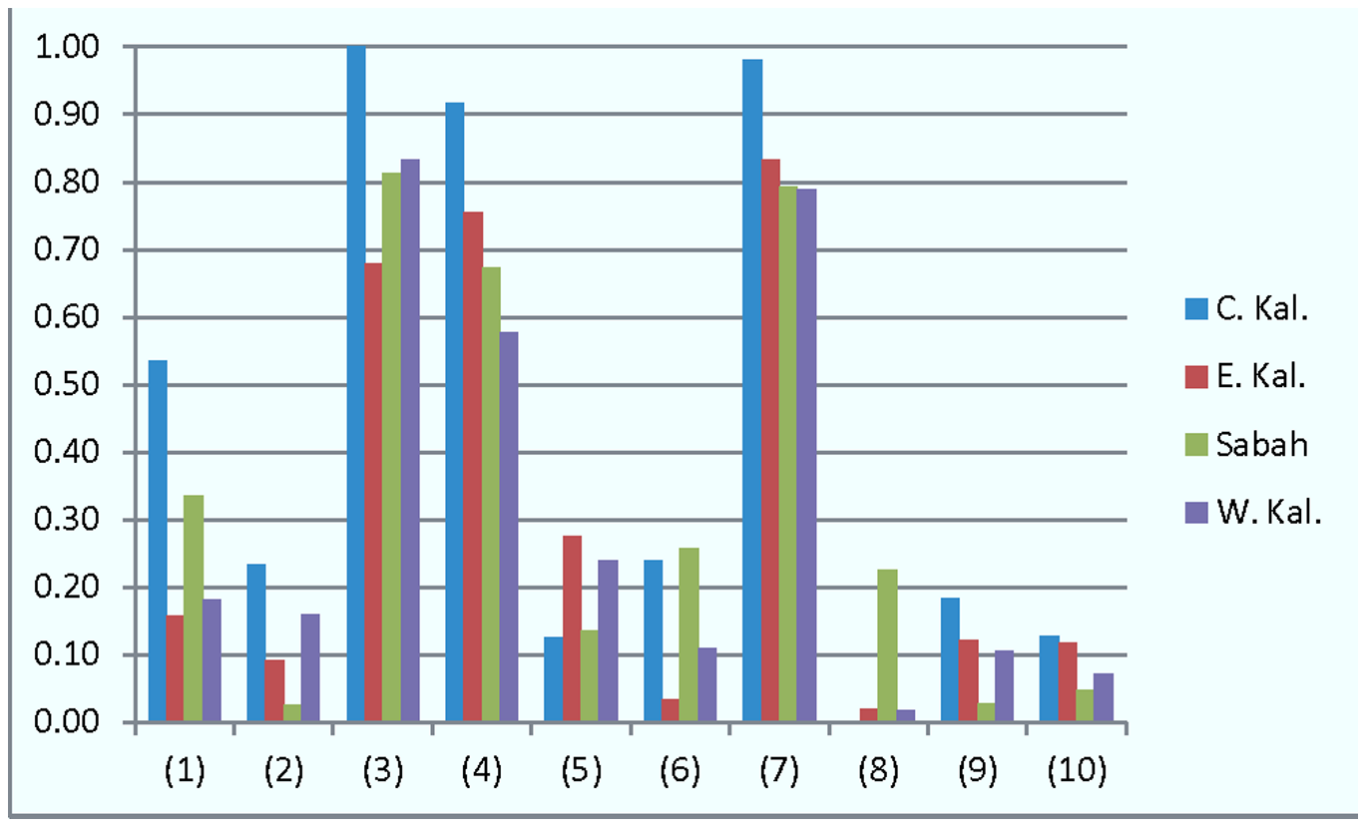
Variation in index scores by geographic region (Province or State). Regions are Central Kalimantan (light blue), East Kalimantan (red), Sabah (green), West Kalimantan (purple). Indices are (1) Direct economic uses, (2) Other forest uses, (3) Cultural and spiritual importance, (4) Importance for health, (5) Environmental health benefits, (6) Direct health benefits, (7) Ecosystem services, (8) Advantages of small-scale clearing, (9) Advantages of large-scale clearing, (10) Disadvantages of large-scale clearing.

The survey respondents were distributed across geographic regions (i.e., Provinces or States herein named as ‘provinces’) as follows: Central Kalimantan (211), East Kalimantan (120), Sabah (72), West Kalimantan (1,434). There was a highly significant difference between provinces for eight of the indices considered in this study; the two indices that were consistent across regions were Importance for health (uniformly high), and Ecosystem services (Figure 6). The largest variation was observed for Direct economic uses (index 1 in Figure 6) with West Kalimantan and Sabah respondents reporting relatively very high (weighted) average scores; Other forest uses (index 2) with Sabah respondents reporting relatively few such uses; and Direct health benefits (index 6) and Advantages of small-scale clearing (index 8) with Sabah reporting relatively more benefits or advantages compared with the other regions. The disadvantages of large-scale clearing (index 10) were more prominently perceived in East Kalimantan compared to the other regions.

### Sensitivity Analysis

As described above the full dataset of responses was inspected carefully for quality by author EM based on within village variation and text length of responses (i.e., did interview teams correctly ask questions and note answers?). The above analyses were restricted to the responses that were coded as high quality. To check for consistency of results, all of the analyses were repeated using the larger dataset of n = 5,410 respondents. As anticipated, this led to small changes in the correlations between the indices and the order of importance of variables associated with each of the indices. However, the corresponding model fits were poorer and the results of the analyses were much more difficult to interpret. This provided support for the decision to confine analyses to the high quality responses. To clarify this further, we assume that because the questions about forest perceptions were at the end of a relative long interview, and that the quality of both questioning and answering may have declined due to fatigue or loss of interest. Our method of eliminating missing or duplicated answers (suggesting that the question may not have been asked at all or once only per village), or very brief ones (suggesting that either the interviewer or interviewee) had lost interest in the question is justified, although we recognize that it may have introduced a potential bias by eliminating interviewees that could not answer the questions about forest perceptions because they never thought of forests.

## Discussion

### Methodological Considerations

During the design phase of the study, we anticipated that the breadth of the sampling regime would require a sacrifice within the interview complexity. However, despite the lack of detailed local information, the study has resulted in a powerful set of insights on forest perception and use patterns over a wide and dynamically complex landscape. Sensitivity analyses using a range of statistical methods showed that simple regression analyses gave poor fits, whereas the substantive BRT and CART analyses showed much stronger fits and explanatory value. This strongly indicates that forest use, perceived benefits of forest and attitudes to small-and large-scale forest clearing are dependent on a complex interaction of individual-level, village-level and contextual land cover variables. The BRT analyses identified sets of variables that were important for each index and the CART analyses illustrated the complexities of interactions between variables. Both the BRT and CART analyses revealed highly nonlinear relationships between the variables and the indices.

Compared with the parametric regression analyses, the nonparametric BRT and CART models produced much better model fit, in terms of correlations between observed and predicted values (for continuous indices) and classification accuracy rates (for binary indices). This was in part expected, given the distributions of the index values (Table S2). The robustness of the estimates and inferences obtained from the BRT and CART models was also strengthened by the stringent fitting criteria used in the analyses (see Methods section).

Our findings mirror those by Sodhi et al. [40] who found remarkably similar responses to ours. Their high response rate may, however, reflect the direct asking of questions about each service, in contrast to our study in which people nominated services unprompted during responses to a broader question of why forests were important. Our indirect way of obtaining information about forest benefits influences the findings. For example, our data indicate that many people volunteered information on floods, air, and other forest services, but any one service was usually mentioned by less than 30% of respondents. This looks fairly small, and we do not really know what the other 70% thought. This is a consequence of how we asked the question, indicating that absences are not informative. The patterns in our study may also reflect the tendency of people to talk about one thing or only a few things in an open question, which can lead to negative correlations between some responses that may actually have a positive association when asked directly, such as number of environmental versus direct health benefits (i.e., maybe people tend to answer about one or the other type of benefits, and then their answer is ‘done’). We believe there is value in the spontaneous reporting of services, as it prevents leading questions with “yes”/”no” answers where it is harder to judge how truthful the answer is. Perhaps it would be ideal if any future surveys asked the open question and then asked directly for people’s views on each of a moderately large set of ecosystem services.

We discussed potential social desirability biases introduced by reporting on illegal activities (e.g., illegal logging or mining) elsewhere [29] and admit that the influence of this on perception patterns is difficult to interpret. Social desirability biases may be negative (leading to under-reporting), possibly due to knowledge of the illegality of certain activities, or positive (leading to over-reporting) if respondents are inclined to boast about these activities or if they perceive that positive responses are related to good skills or knowledge of the forest. We suggest that future survey employ randomised response techniques, like those recently trialled to assess illegal fly fishing in Wales [41], or anonymous self-completion of questionnaires, which has also been shown to reduce social desirability bias in some contexts [42].

Finally, despite the methodological strengths of these analyses, there was inherent spatial bias from the study design. Notably, the sampling frame in the primary surveys was principally driven by orangutan presence thus producing a non-random sample of vegetation types, social and cultural variation and land uses. We sampled about one third of Borneo’s land area, and extrapolation from the sampling frame to other parts of the island needs to keep that spatial bias in mind. An additional bias could be gender-related, since 20.5% only of the 1,837 respondents included in the analyses were female. We discuss the implications of these and other biases elsewhere [29], [30], but remind readers that the perceptions reported here are primarily those of men living in landscapes with moderate to high levels of forest cover, within the range of orangutans.

### Notable Perception and use Patterns

Data in Text S1 indicate that among forest uses, timber, rattan, bushmeat and fish, fire wood, traditional medicine, and forest gardens stand out as most frequently mentioned. A large variety of other uses were also reported, some of which can be of high local economic importance (e.g., artisanal gold mining and harvest of aloes wood) and generally accord more detailed studies of forest-based livelihoods in Borneo (e.g., [33], [43], [44]). Interestingly, we note the importance of rattan farming within our study, which contradicts prior suggestions that Indonesia’s rattan industry is in decline [45] and that people are not attending their rattan gardens. It might be that rattan is considered a fall-back resource, presently of limited value but with the potential to generate income when this is needed [46].

The majority of respondents (67%) perceive the forest to be very important for their health through provision of medicinal plants and other services: forest is generally perceived as a health giver. In a global review of the link between forests and health, Colfer et al. [47] similarly found that tropical forests provide essential foods, medicines, health care and meaning to peoples all over the world–with benefits generally increasing with proximity to forest. Deforestation and forest degradation have also been associated with increased incidence of infectious diseases [48]. However, relationships between forests and health are complex and developments that accompany deforestation can also bring significant health benefits, at least to some sections of society [47]. This complexity is reflected in the different environmental and social factors that are most strongly associated with forest-health relationships. Our results show that perceptions regarding health benefits from forests are particularly strong among older community members, while younger members may have lower awareness or place less value on traditional medicines or environmental benefits provided by forests (also see below).

Unexpectedly, the most frequently reported environmental benefit was that forests kept the environment cool (33% of respondents). Associations between deforestation and changes in temperature and precipitation have primarily been modelled at regional scales, in the context of improving predictive climate models [49]–[54]. Deforestation is expected to lead to temperature increases due to loss of reflectance and absorption of solar radiation by the canopy and changes in evaporative cooling, but the spatial range of these effects or their consequences for human health have rarely been investigated. We are aware of only one other study that has documented similar perceptions, specifically showing that regulation of temperature and precipitation was perceived as an ecosystem service by 70–95% of households living near five forested protected areas in South East Asia [40]. This indicates that exposure to higher temperatures may be an important impact on communities from loss of nearby forests, and calls for further investigation, especially in relation to the role of forests and trees in adaptation to climate change [55]. Respondents frequently associated increased temperature with higher risk of disease, and also associated forest loss with increased flooding with higher incidence of malaria. Further research is needed to address these potential links between deforestation, local and regional climate change, and disease, in view of their significant consequences for human welfare [56].

The positive perception of forest as sources of clean water, air and oxygen and for prevention of floods, erosion, and landslides accords with biophysical studies of forest landscapes [57], [58], and with interview-based studies of communities near protected areas in South East Asia [40]. Relationships between deforestation and negative environmental impacts are not always simple [58], [59], and in-depth studies are needed to determine whether people’s perceptions about forest services are based on experience or external factors such as media that link deforestation with floods, erosion, and landslides. One noteworthy result from our interview surveys was that only 13 respondents (none in the set of higher quality interviews) mentioned the carbon sequestration functions of forest, although 61 gave “preventing global warming” as a reason for forest benefits. Note that these interpretations should be treated with some caution, since the responses are unprompted, based on an open-ended question; hence it is possible that more people were aware of these issues but not mention them or viewed them as irrelevant to their personal health. Moreover, we recognize that we did not ask respondents about their specific views on global or regional ecosystem services, or indirect benefits via payments for benefits received by others, but that these were unprompted responses regarding why forests are important for their health and the health of their families. Still, the infrequent mention of carbon stands out compared to similarly intangible services such as clean air/oxygen which was mentioned by 857 respondents. Carbon sequestration functions of forests are high on the political agendas of tropical forests countries, especially those which have received international funding to develop programs for reducing carbon emissions through avoided deforestation and forest degradation (REDD) [60], [61]. However, it appears that few local people see the potential of forests to store carbon as important for their health or livelihoods, and that information on potential income or livelihood benefits from carbon-related initiatives has either not reached or not convinced Borneo’s communities. For those trying to implement forest carbon strategies this could either mean that more investments are needed in carbon education, or that the development of carbon projects could piggy-back on the strong perceptions that people have about other services.

Finally, we note interesting patterns in perceptions about forest clearing. Generally people support clearing if it occurs on a small scale and for their own direct use (mostly farming) with 48% of respondents reporting that such clearing is good for their own agricultural purposes. There is much less support for large-scale clearing, with twice as many negative impacts of large-scale deforestation being reported as positive ones (1,187 vs 584 responses, from 46% vs 25% of respondents, see Text S1). Large-scale clearing is perceived by some respondents as providing opportunities for income or employment (14%), land claims and compensation (4%), or improved infrastructure (2%), and our BRT analysis showed that is most likely within villages near established oil palm plantations. On the other hand, more respondents considered large-scale clearing as incurring higher costs than benefits, especially with regard to insufficient employment or other benefits from companies (voiced by 29% of respondents), negative environmental impacts (19%), and reduction in products that communities could obtain from forests (10%). Such insights are useful at national and local government levels were it is decided which lands should be allocated to plantation development. Seeking out lands where local people are most receptive to such plantations could significantly reduce social conflicts.

### Spatial Variation in Perceptions

Regional differences were apparent in levels of support for small-scale clearing or lack of support for large-scale clearing. Positive views of small-scale clearing were generally higher in West Kalimantan and especially in Sabah. Negative views of large-scale clearing were most common in East, West and Central Kalimantan, and less frequent in Sabah. Official government data suggests that, between 2000 and 2010, natural forest cover (excluding planted forest) in West Kalimantan declined from 51.1% to 45.9%, in Central Kalimantan from 64.6% to 51.3%, in East Kalimantan from 71.6% to 66.4% [62], [63], and in Sabah from about 58.5% to 51% [64]. This indicates that respondents in the regions with lowest current forest cover and annual forest loss (W. Kalimantan and Sabah), and with a longer history of deforestation compared to other parts of Borneo [65], [66], tended to see small-scale clearing as beneficial, and were least opposed to large-scale deforestation. Respondents from areas with the highest current forest cover (East Kalimantan) or recent forest loss (Central Kalimantan), were the most frequently opposed to large-scale deforestation. These patterns suggest that deforestation initially results in negative perceptions about its impacts, and that in areas where non-forest land uses have become established over longer periods, there are more mixed views of the benefits and disadvantages. A follow-up study in which we have translated the present statistical models into spatial outputs (N. Abram et al. unpubl. data) actually shows a more complicated picture. Areas presently undergoing deforestation appear to have reduced forest perceptions. This might be because local people can benefit significantly from the deforestation process, either by their involvement in timber harvest, sale of land or land use, or development of new infrastructure. These people appear to be less concerned about the loss of forest services than people in remote forest areas. Environmental benefits from forests (e.g., flood prevention) are considered particularly important in areas where deforestation happened decades ago, possibly because people were most strongly affected by negative environmental impacts.

### The Importance of Interpreting Villagers’ Perceptions

To our knowledge, this is the first study that describes the factors that influence peoples’ perceptions and use values of forests and provisioning services and benefits across a large, transnational tropical landscape. The design of our study allowed us to address a range of forest values that are often omitted during forest valuation studies based on detailed household surveys (after [15]): e.g., forest products that are not sold in markets; wood used for construction; insurance values of forests, especially for poor people; hydrological services, protective values of forests and other environmental benefits. Our approach provides insights into broad scale patterns of forest use and the forest-related factors that influence people’s well-being. Many social scientists involved in detailed livelihood and household studies of forest communities might consider our study too general in design to say anything meaningful about any particular community. We agree that even though that might be the case, the strength of our approach lies exactly in these generalizations, and their supporting statistical models. These could assist the development of forest policies that can specifically target people in particular areas of high forest use and valuation. For example, an improved understanding of how forest conversion and deforestation could affect people that depend on forest resources could facilitate more optimal land use planning and therefore reduce social conflict. Our models highlight certain regions of Borneo where the forest is strongly associated with people’s well-being and survival. Development of these areas should take into consideration how much people depend on forest products (building materials, food, medicine, etc.) and value the forest benefits in terms of general health and welfare, and avoid reducing access to these forests.

Interestingly, our surveys suggest that most people in Borneo still use “forest” resources even in areas where, technically speaking, no more closed-canopy forest remains. This indicates the importance of even heavily degraded forest stands for people’s livelihoods, suggesting that the common treatment of such degraded lands as “useless” and only suitable for conversion to non-forest uses such as oil palm plantations [67] is not necessarily warranted [68]. From personal experience we know that people highly value remnant forest stands even when embedded in a matrix of industrial plantations for a range of direct utilitarian (e.g., timber, fruit), indirect (e.g., clean water) and other reasons (spiritual, old village sites, traditional graveyards). Such forest fragments can also be highly valuable for wildlife if factors such as over-exploitation are controlled [69], [70].

The patterns described in this paper are increasingly recognized by Borneo’s governments and developed into policies that take people’s forest use requirements into consideration. For example, Indonesia has been piloting a range of new community land use title policies (e.g., *Hutan Hak*, *Hutan Adat*, *Hutan KeMasyarakatan*, *Hutan Desa*, and *Hutan Tanaman Rakyat*, see [71]), and Sabah has initiated several community-based initiatives, such as the “Sustainable Community Forest Management Program” [72]. Testing and implementation of these land use policies has, however, been slow [61]. One promising movement in this regard are recent government commitments to accelerate legal recognition of community-based forest management [73], which could be one step closer towards secure tenure. Our study could further facilitate these government processes by pointing out areas where forest dependence and perceived values are highest. This would be especially useful if forest perception patterns could be translated into spatial maps with continuous coverage –a process that we are developing in a concurrent study within the Borneo Futures initiative. Such spatial representations of forests important for communities could facilitate informed land use planning and zoning in areas of high social or cultural importance, especially if we can learn more about variation in perceptions with gender, age and landscape contexts. The information could help government decision-makers to optimize the balance between generating revenues from forest exploitation and the needs to sustain future production and ecosystem services, and minimize negative environmental and social impacts. Such approaches are especially important for longer-term strategic planning, such as Indonesia’s Master Plan for Kalimantan [74]. Further studies such as this are vital if we believe that the people of Borneo should have a voice in the future of the landscapes they live in, and if governments are truly committed to incorporating local needs and aspirations into their decisions.

## Supporting Information

Table S1 **Classes of LULC, descriptions and processing steps.**
(DOCX)

Table S2 **Statistical summaries of the Forest Perception indices.**
(DOCX)

Text S1 **Summary of responses in decreasing order of importance from 1,837 most reliable respondents.**
(DOCX)
