# Supplementary material for: People’s Perceptions about the Importance of Forests on Borneo
Source: PLoS One. 2013 Sep 9;8(9):e73008. doi: 10.1371/journal.pone.0073008 (PMC3767661; doi:10.1371/journal.pone.0073008)
Supplement: Table S1 — Classes of LULC, descriptions and processing steps. (DOCX) [file pone.0073008.s001.docx]

People’s perceptions on the importance of forests on Borneo

Table S1. Classes of LULC, descriptions and processing steps.

| Class | Description | Processing steps |
| --- | --- | --- |
| 1. Intact natural forest | Medium to tall old-growth natural forests that have never been logged by the timber industry. Open to closed canopy: closure is probably higher than 30%. Includes Lowland and montane dipterocarp forests, riverine forests, heath forests on plateaux, tall closed-canopy peat forests and open-canopy pole peat forests. Note, our intact forest class may include areas where the forest has been degraded slightly by small-scale logging, which we could not detect using Landsat imagery. | Step 1: Generated a forest cover map for year 2010 using SarVision 2010 land cover map. Merged classes: 3 (pole peat forest), 6 (closed forest), 11 (riparian forest) and 17 (peat-swamp forest close canopy).  Step 2: transformed the 1990-2000-2010 logging road network (indicating mechanized logging) into a road density index (km/km^2^) of 1x1km grid cell (search radius 5km).  Step 3: overlaid the road density map onto the forest cover map generated in Step 1 and recoded forest into intact in areas where road density = 0. Areas where road density > 0 were coded as class 9 (see below). |
| 2. Mangroves | Closed canopy Medium Forest with closed canopy of 10 % to 30% occurring in tidal affected zones. | This class has been taken directly from SarVision 2010 land cover map. |
| 3. Agroforests / forest regrowth | Medium to tall agro-forests and forest regrowth. Open to closed canopy: closure is probably equal or higher than 30%. Includes traditional rubber agroforests, fruit gardens, and land under fallow, where forests is regenerating. | Step 1: Generated a broad vegetation cover map including agroforests/forest regrowth/very degraded logged forests using SarVision 2007 and 2010 land cover map. Merged class 8 (forest mosaic, fragmented or degraded forest) from SarVision’s 2007 land cover map with class 2 (woodland) and class 13 (open forest) from SarVision’s 2010 land cover map.  Step 2: Overlaid the logging road network onto this broad vegetation map and recoded this class into agroforests/forest regrowth in areas where there were no logging roads. |
| 4. Non-forest | Includes: 1) Low vegetation of grasses or shrubs occurring on drained soils, occasionally flooded; 2) dry rice cultivation; 3) Low herbaceous vegetation with including tall grasslands and ferns; 4) can include agricultural cropland areas; 5) dry to occasionally flooded terrain; 6) areas of herbaceous vegetation, 7) shrub lands and young forest regrowth in fallow lands. | This class has been generated by merging classes 4, 5, 7, 8, 9, 10, 15 from SarVision’s 2010 land cover map. |
| 5. Water bodies | Large lakes and large rivers. | As identified up by SarVision’s 2010 land cover maps |
| 6. Oil palm plantations in 2010 | Planted or recently cleared industrial scale oil palm plantations as of year 2010. | Industrial Oil palm plantations in 1990-, 2000-, and 2010-eras were manually digitized in ArcGIS 10 by visual inspection of >150 Landsat satellite images downloaded from the Global Land Survey database (http://earthexplorer.usgs.gov/). Industrial-scale plantations were readily identified as large geometrically-shaped areas with distinctive homogeneous spectral signatures characteristic of monoculture stands. We digitized any area planted with or being cleared for oil palm. Imagery acquired at earlier dates from the main key dates were often required to verify clearing and planting because newly cleared plantations (<1yr since planting) is usually easiest to detect using Landsat imagery. |
| 7. Industrial timber plantations | Planted or recently cleared industrial scale timber plantations as of year 2010. | Industrial Oil palm plantations in 1990-, 2000-, and 2010-eras were manually digitized in ArcGIS 10 by visual inspection of >150 Landsat satellite images downloaded from the Global Land Survey database (http://earthexplorer.usgs.gov/). Industrial-scale plantations were readily identified as large geometrically-shaped areas with distinctive, homogeneous spectral signatures characteristic of monoculture stands. We digitized any area planted with or being cleared for rubber or *Acacia mangium*). Imagery acquired at earlier dates from the main key dates were often required to verify clearing and planting because newly cleared plantations (<1yr since planting) is usually easiest to detect using Landsat imagery. |
| 8. severely degraded logged forests | This class includes natural old-growth forests that have become so severely degraded that they no longer resemble the spectral signatures of forests in class 1 or 9. These forests are primarily found in east Kalimantan, and elsewhere only occur in small areas in Sabah, Sarawak and south Kalimantan. In east Kalimantan these forests have been burnt severely twice in March-April 1983 and March-April 1998 (i.e. during the two most intense El-Niño fire pulses on record, also declared national disaster in Indonesia). This forest class shows little sign of regenerating towards tall forest, probably because of invasion by flammable grasses. | Step 1: Similar to the process for generating class 4, we first generated a broad vegetation cover map including agroforests/forest regrowth/very degraded logged forests using the SarVision 2007 and 2010 landcover map. Merged class 8 (forest mosaic, fragmented or degraded forest) from SarVision’s 2007 land cover map with class 2 (woodland) and class 13 (open forest) from SarVision’s 2010 landcover map.  Step 2: Overlaid the logging road network onto this broad vegetation map and recoded this class into severely degraded logged forests in areas where there were logging roads. |
| 9. Logged forests | Medium to tall old-growth natural forests that have been logged by the timber industry using heavy machinery and networks of logging trails. Open to closed canopy: Includes Lowland and montane dipterocarp forests and tall closed-canopy peat forests. | Step 1: Generated a forest cover map for year 2010 using SarVision 2010 landcover map. Merged classes: 3 (pole peat forest), 6 (closed forest), 11 (riparian forest) and 17 (peat-swamp forest close canopy).  Step 2: transformed the 1990-2000-2010 logging road network (indicating mechanized logging) into a road density index (km/km^2^) of 1x1km grid cell (search radius 5km).  Step 3: overlaid the road density map onto the forest cover map generated in Step 1 and recoded forest into logged forests in areas where road density > 0. (If road density = 0, see class 1). |
