# Supplementary material for: People’s Perceptions about the Importance of Forests on Borneo
Source: PLoS One. 2013 Sep 9;8(9):e73008. doi: 10.1371/journal.pone.0073008 (PMC3767661; doi:10.1371/journal.pone.0073008)
Supplement: Table S2 — Statistical summaries of the Forest Perception indices. (DOCX) [file pone.0073008.s002.docx]

Table S2: Statistical summaries of the Forest Perception indices. 1^st^ quartile, mean, 3^rd^ quartile and standard deviation for each of the ten forest perception indices: (1) Direct economic uses, (2) Other forest uses, (3) Cultural and Spiritual Importance, (4) Importance to health, (5) Environmental health benefits, (6) Direct health benefits, (7) Ecosystem services, (8) Advantages of small scale clearing, (9) Advantages of large scale clearing, (10) Disadvantages of large scale clearing. All indices are scaled to the range 0-1 .

| Index | (1) | (2) | (3) | (4) | (5) | (6) | (7) | (8) | (9) | (10) |
| --- | --- | --- | --- | --- | --- | --- | --- | --- | --- | --- |
| 1^st^ Qu. | 0.20 | 0.00 | 0.00 | 0.00 | 0.00 | 0.00 | 0.50 | 0.00 | 0.00 | 0.00 |
| Mean | 0.38 | 0.17 | 0.74 | 0.92 | 0.24 | 0.14 | 0.77 | 0.11 | 0.11 | 0.09 |
| 3^rd^ Qu. | 0.60 | 0.33 | 1.00 | 1.00 | 0.25 | 0.33 | 1.00 | 0.20 | 0.33 | 0.14 |
| s.d. | 0.25 | 0.16 | 0.44 | 0.26 | 0.21 | 0.19 | 0.26 | 0.16 | 0.19 | 0.15 |
