# Supplementary material for: People’s Perceptions about the Importance of Forests on Borneo
Source: PLoS One. 2013 Sep 9;8(9):e73008. doi: 10.1371/journal.pone.0073008 (PMC3767661; doi:10.1371/journal.pone.0073008)
Supplement: Text S1 — Summary of responses in decreasing order of importance from 1,837 most reliable respondents. (DOCX) [file pone.0073008.s003.docx]

People’s perceptions on the importance of forests on Borneo

Text S1. Summary of responses in decreasing order of importance from 1,837 most reliable respondents. Number between brackets indicate number of respondents who gave a particular answer.

Direct economic uses: Timber (1234); rattan (950); hunting (831); traditional medicine (624); mining (245); honey (342); aloes wood (303). Other Forest Uses: Fish (368); fire wood/small wood (351); forest gardens (264); rubber (157); fruit and vegetables (234); tree sap (*damar*/*jelutung*) (95); illipe nuts (*Shorea* spp.) (65); passing through and survey (47); bark of *Nothaphoebe coriacea* and *N. umbelliflora* (45); oil palm (37); employment (25); fruits from *Litsea* spp. (25); spiritual values and enjoyment (23); *Mitragyna speciosa*, leaves for medicine (16); non-timber (general) (15); edible swift nests (13); food (9); source of water (9); nipa (*Nypa fructicans*) and other roofing material (7); back up resource (6); binding materials (5); orchids and ornamental plants (5); bamboo (4); tourism (1); place for rare fauna, conservation, and research (6); prawns (3); sugar palm (1). Importance of Forest for Health: Very important (1236); quite important (461); not important (40); don’t know (98). Direct health benefits: Medicine (282); general welfare, life giver, and for daily needs (252); spiritual value, people-nature association (85); forest prevents disease (e.g., malaria in flooded areas) (77); place to live and forest protects the village from disasters (25); good for planting (12); for future generations (11); economic use and gardens (11); good soil (7); tourism and relaxation (3); source of electricity (hydropower) (1). Environmental health benefits: Cool and shaded from sunshine (605); source of water (342); clean air and oxygen (275); flood prevention (219); prevent pollution (98); environmental protection and species habitat (81); storm reduction and climate control (39); erosion prevention (36); landslide prevention (29); global warming (15); carbon sequestration (4); fire prevention (3); ozon protection (2); longer or more severe dry season and droughts (2). Cultural and Spiritual Benefits: very significant (871); quite significant (484); insignificant (404); don’t know (76). Advantages of small-scale forest clearing: For forest gardens and agricultural fields (368); for agricultural crops (198); for rubber (to plant it) (184); for rice cultivation (71); for local community, company clearing not good (51); for oil palm (to plant it) (49); for timber and fire wood (33); for fruit and vegetables (18); for building or a place to live (13); for other forest products (5). Advantages of large scale clearing: For family needs, a source of income, and community welfare (259); for work or business (202); to own land, expand land, or claim land (72); for infrastructure and other help from companies (school, scholarship, clean water, electricity) (30); for getting land compensation or logging payment (5); for mining (5); it is good as long as it is legal (4) ; for better hunting and fishing on cleared land (4); because forest will never be finished and it will grow back (3) because forest needs to be used (2). Disadvantages of large scale clearing: Deforestation doesn't benefit communities enough, they do not provide enough work, and communities suffer (259); negative environmental impacts from logging (floods, temperature, erosion) (199); companies don't provide enough work or other community benefits and companies benefit more than people (147); fewer forest products, including timber (122); oil palm and other plantations provide insufficient benefits (121); protection of forest needed and deforestation destroys nature (86); there is not enough forest left and deforestation will destroy remaining forest (71); affects our future needs (44); clearing reduces available land for communities and clearing destroys gardens (28); forest better for hunting and fishing, and deforestation reduces wildlife (21); once the forest is gone there is no more work (19); newcomers or outsiders benefit more than local communities (14); only temporary benefits from deforestation but long term losses; forest should be sustainably managed (10); not everyone benefits from forest clearing (8); companies manage the forest badly and companies lie (8); it is our forest, why would anyone else clear it (8); not everyone benefits from forest clearing (8); it doesn't directly make land suitable for agriculture (4); it customary law prohibits it, and its illegal (4); it increases pests (2); we do not get enough compensation (2); it is better if communities manage the forest (1); no money to invest and therefore we cannot benefit (1).
